# Development of a computerised decision aid for thrombolysis in acute stroke care

**DOI:** 10.1186/s12911-014-0127-1

**Published:** 2015-02-07

**Authors:** Darren Flynn, Daniel J Nesbitt, Gary A Ford, Peter McMeekin, Helen Rodgers, Christopher Price, Christian Kray, Richard G Thomson

**Affiliations:** Institute of Health and Society, Newcastle University, Baddiley-Clark Building, Richardson Road, Newcastle upon Tyne, UK; School of Computing, Newcastle University, Newcastle upon Tyne, UK; Institute for Ageing and Health (Stroke Research Group), Faculty of Medical Sciences, Newcastle University, Newcastle upon Tyne, UK; Wansbeck General Hospital, Northumbria Healthcare NHS Foundation Trust, Ashington, UK; Institute for Geoinformatics, University of Münster, Münster, Germany

**Keywords:** Decision support, Decision aid, Patient information, Shared decision making, Risk communication, Thrombolysis, Acute stroke

## Abstract

**Background:**

Thrombolytic treatment for acute ischaemic stroke improves prognosis, although there is a risk of bleeding complications leading to early death/severe disability. Benefit from thrombolysis is time dependent and treatment must be administered within 4.5 hours from onset of symptoms, which presents unique challenges for development of tools to support decision making and patient understanding about treatment. Our aim was to develop a decision aid to support patient-specific clinical decision-making about thrombolysis for acute ischaemic stroke, and clinical communication of personalised information on benefits/risks of thrombolysis by clinicians to patients/relatives.

**Methods:**

Using mixed methods we developed a COMPuterised decision Aid for Stroke thrombolysiS (COMPASS) in an iterative staged process (review of available tools; a decision analytic model; interactive group workshops with clinicians and patients/relatives; and prototype usability testing). We then tested the tool in simulated situations with final testing in real life stroke thrombolysis decisions in hospitals. Clinicians used COMPASS pragmatically in managing acute stroke patients potentially eligible for thrombolysis; their experience was assessed using self-completion forms and interviews. Computer logged data assessed time in use, and utilisation of graphical risk presentations and additional features. Patients’/relatives’ experiences of discussions supported by COMPASS were explored using interviews.

**Results:**

COMPASS expresses predicted outcomes (bleeding complications, death, and extent of disability) with and without thrombolysis, presented numerically (percentages and natural frequencies) and graphically (pictographs, bar graphs and flowcharts). COMPASS was used for 25 patients and no adverse effects of use were reported. Median time in use was 2.8 minutes. Graphical risk presentations were shared with 14 patients/relatives. Clinicians (n = 10) valued the patient-specific predictions of benefit from thrombolysis, and the support of better risk communication with patients/relatives. Patients (n = 2) and relatives (n = 6) reported that graphical risk presentations facilitated understanding of benefits/risks of thrombolysis. Additional features (e.g. dosage calculator) were suggested and subsequently embedded within COMPASS to enhance usability.

**Conclusions:**

Our structured development process led to the development of a gamma prototype computerised decision aid. Initial evaluation has demonstrated reasonable acceptability of COMPASS amongst patients, relatives and clinicians. The impact of COMPASS on clinical outcomes requires wider prospective evaluation in clinical settings.

**Electronic supplementary material:**

The online version of this article (doi:10.1186/s12911-014-0127-1) contains supplementary material, which is available to authorized users.

## Background

Thrombolysis (the breakdown of blood clots using pharmacological agents; commonly called ‘clot-busting drugs’) administered within 4.5 hours of acute ischaemic stroke onset (caused by a sudden blockage of an artery supplying blood flow to, or within, the brain) improves outcome [1]. However, thrombolytic treatment can cause bleeding complications, the most serious being symptomatic intracranial haemorrhage (SICH) that typically occurs within 24–36 hours and leads to clinical deterioration or death [2,3]; although 90 day mortality is not increased in patients treated with thrombolysis [4]. Efficacy is time dependent, with earlier treatment associated with increased likelihood of functional independence (complete recovery or minor disability) after acute stroke [4,5].

The thrombolysis decision-making context (extreme time dependent nature of treatment outcome, and the need to rapidly consider the trade-offs between the likely long-term benefit and early risk of SICH and its consequences) presents unique challenges for clinicians, patients and their relatives or proxy [6].

Aggregate-level estimates of the likely balance of benefits and risks of harm from treatment derived from event rates reported in randomised controlled trials [4,5] and patient registries [7,8] have been used to support clinical decision-making about thrombolytic treatment and to convey probabilistic information on outcome states to patients/relatives. However, benefit-to-harm ratios differ as a function of individual patient characteristics due to variation between patients who fulfil the licensing criteria for treatment. The weighing up of value in treating any individual patient and communication of this complex information (alongside eligible patients presenting too late to secondary care and lack of adequate infrastructure to support delivery of thrombolysis services [9,10]) is a key reason why thrombolysis is an under-utilised treatment for acute stroke and door to needle times (arrival time at hospital to administration of thrombolysis) are sub-optimal [11,12]. Additional factors inhibiting the use of thrombolysis include physician-related factors such as uncertainty about effectiveness, apprehensions about increased risk of SICH, and unresolved issues on relative contraindications for treatment [5,13-15], and lack of robust data on the likely balance of benefits and risks of treatment in routine practice as a function of individual patient characteristics [16].

Evidence-based tools for thrombolysis in acute stroke such as decision aids [17] are warranted to (i) optimise treatment rates by assisting clinicians to weigh-up the potential net benefit in treating any individual patient; (ii) support clinicians in communicating accurate information on risks/benefits and prognosis to patients (or next of kin/proxy); and (iii) seamlessly support different approaches to decision-making about thrombolysis, including (where appropriate) engagement of patients/relatives in shared decision-making with stroke clinicians [6,18]. However, a recent review identified sub-optimal development (e.g., lack of testing in clinical settings) and content (e.g., failure to convey balanced synopses of benefits/risks) of decision support, patient information and risk communication tools for thrombolysis in acute stroke [6].

The thrombolysis decision-making context in acute stroke care may be viewed as one in which both clinicians and patients/relatives will gravitate toward a paternalistic model of decision-making. However, the optimal approach to decision-making in emergency contexts such as acute stroke may vary on a case-by-case basis, and stroke clinicians are best placed to facilitate the engagement of patients or their relatives/proxy in a thrombolysis shared decision-making process as much as they desire, as appropriate, in accordance with their preferences and values [19]. Indeed, the decision to treat acute stroke with or without thrombolysis represents a choice-based decision under conditions of uncertainty involving trade-offs between the likely benefit and risk of harm, which is sensitive to the preferences and values of patients with regards to treatment and likely outcome states following acute stroke [20-22]. These conditions are appropriate for shared decision-making.

Exploratory work (interviews with 37 patients/relatives and with 23 clinicians involved in decision making and information provision about thrombolysis) has been reported elsewhere [3]. In summary, this revealed a need: to strengthen relational (face-to-face) decision support from clinicians to guide patients/relatives through the hyper-acute stroke period and thrombolysis decisions; and for decision support for clinicians to weigh-up the value in treating any individual patient with thrombolysis and for communicating individualised benefits/risks to patients/relatives.

As self-report obtained from interviews does not always equate to actual practice, we also used ethnographic methods, including participant observation and informal discussions to explored decision-making processes and practices in situ in three acute units in the north east of England. Participant observation [129.5 hours] enabled examination of the way in which individuals organised and made sense of their experiences, whilst informal discussions provided clarification. Data analysis drew on principles of the constant comparative method. Evolution of field notes and coding were undertaken iteratively and concurrently with further data collection. After multiple readings of the data, categories and codes were derived either directly from the data in the terms used by participants, with reference to relevant literature.

Analysis of seven thrombolysis assessment/decision-making interactions between patients/relatives and clinicians in three acute stroke units revealed clinicians’ had variable preferences on the ‘‘right time” to raise the possibility of thrombolytic treatment with patients/relatives (i.e. before or after CT brain imaging). This reinforced the need for rapid and pragmatic decision support that would be accessible across the acute stroke pathway. Detailed findings of this phase are available from the corresponding author.

Following a structured process and based on published guidance [23,24], our objectives (informed by our initial exploratory work) were to: (i) establish the optimal mode and content of a decision aid to support eligibility decision-making about thrombolysis for individual patients; and clinical communication of personalised information on the benefits and risks of thrombolysis to patients/relatives to support different approaches to decision making in the acute stroke clinical setting; (ii) identify and describe the key components of a resultant prototype of a **COMP**uterised decision **A**id for **S**troke thromboly**S**is (COMPASS); and (iii) establish the usability of the prototype decision aid with clinicians and patients/relatives, in order to refine the user interface and information content to enhance its acceptability and feasibility in the acute stroke clinical setting.

## Methods

**Figure 1:**
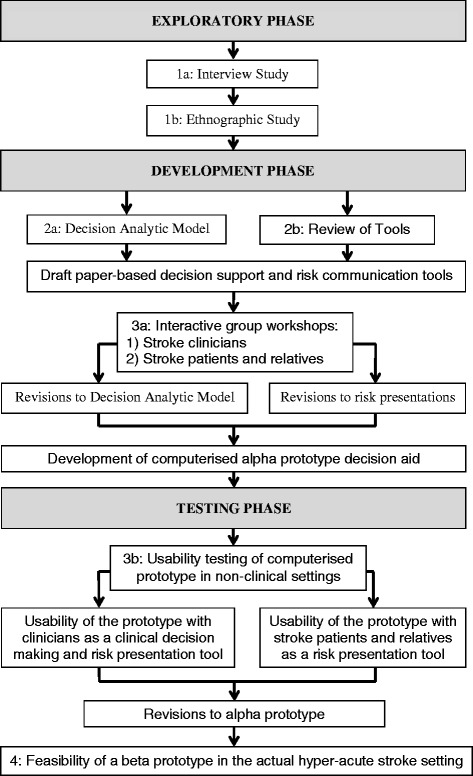
**Overview of the development process.**

A synopsis of the development process is shown in Figure 1. Ethical and research governance approval for each phase (where required) was secured from Research Ethics Committees and participating Hospital Trusts. Written informed consent was obtained from clinicians and patients/relatives.

### Development phase

Informed by exploratory work, the aims of this phase were to (i) develop a robust decision analytic model (DAM) to calculate predictions for acute stroke outcomes (e.g., death and extent of disability) as a function of individual patient characteristics; and (ii) identify the optimal mode of delivery (paper-based or electronic), form (numerical or graphical risk presentations to convey outcome probabilities derived from the DAM) and content (language to convey key information such as descriptors for outcome states and time horizons for outcome probabilities) of a prototype decision aid for thrombolytic treatment.

#### Decision-analytic model (DAM)

The development process for the DAM to predict the patient-specific probability of acute stroke outcomes is reported in detail elsewhere [25], Briefly, the predictive equations within the Stroke-Thrombolytic Predictive Instrument [S-TPI] [26] were used as a basis to construct the DAM. The S-TPI enables patient-specific predictions at three months, with and without thrombolysis, for a normal/near normal outcome (defined as a modified Rankin Scale (mRS) ≤1, which equates to no symptoms or slight disability - as a function of seven patient variables); and a catastrophic outcome (defined as a mRS ≥ 5, which equates to severe disability/death - as a function of three patient variables).

There are the differences between predicted outcomes from the S-TPI and actual outcomes in routine clinical practice [27,28]. Therefore using data from 2,401 routinely treated stroke patients from the Safe Implementation of Thrombolysis in Stroke UK database [7] the original S-TPI predictive equations were adjusted to ensure: (i) consistency between outcomes predicted by the DAM and actual outcomes of patients treated in routine practice; and (ii) that definitions of outcomes were representative of those typically used in clinical practice (functional independence [mRS 0 to 2] - complete recovery/minor disability; dependence [mRS 3 to 5] – moderate/severe disability, and death); and (iii) the inclusion of additional predictors of functional independence from observational studies of patients treated in routine practice [29]^.^ Predictions in the DAM for mRS 0 to 2, 3 to 5 and death in untreated patients were validated using untreated patient data (N = 5,715) from the Virtual International Stroke Trials Archive [30].

A scoring model derived from patients treated with thrombolysis in routine practice [31]) was selected to calculate patient-specific predictions of risk of SICH. A suitable predictive equation for outcomes following SICH could not be identified in the literature. Therefore, the subsequent impact of SICH on outcomes at three months used proportions of patients that would likely be mRS 0 to 2, 3 to 5 and dead following SICH [32].

#### Interactive group workshops

A suite of draft paper-based tools (Additional file 1) were developed to convey the outcomes generated by the DAM (informed by a literature review of currently available tools, published elsewhere [6] and guidance on presentation of outcome probabilities [33]) to support eligibility decision making about thrombolysis for individual patients (structured look-up tables and tables of decisions rules for different levels of net benefit from thrombolysis) and clinical communication of personalised information on the risks/benefits of thrombolysis to patients/relatives (clustered and stacked bar graphs, pictographs and flowchart diagrams).

Draft paper-based tools were presented within interactive workshops (mixture of demonstration, open discussion and small group exercises) with 12 stroke clinicians (five stroke physicians, two emergency department physicians, five stroke nurses) and with eight patients with a history of previous stroke, and seven of their relatives. Field notes on salient points and reactions of the participants were recorded, and summarised for discussion within the research team to inform the development of an alpha prototype of the decision aid for usability testing.

#### Development of alpha prototype

One of the authors (DN), a computing science graduate with six years of programming experience (with support from a senior computing scientist, CK) developed the software, spending approximately 10 weeks [full-time hours] to develop the alpha prototype of COMPASS.

**Figure 2:**
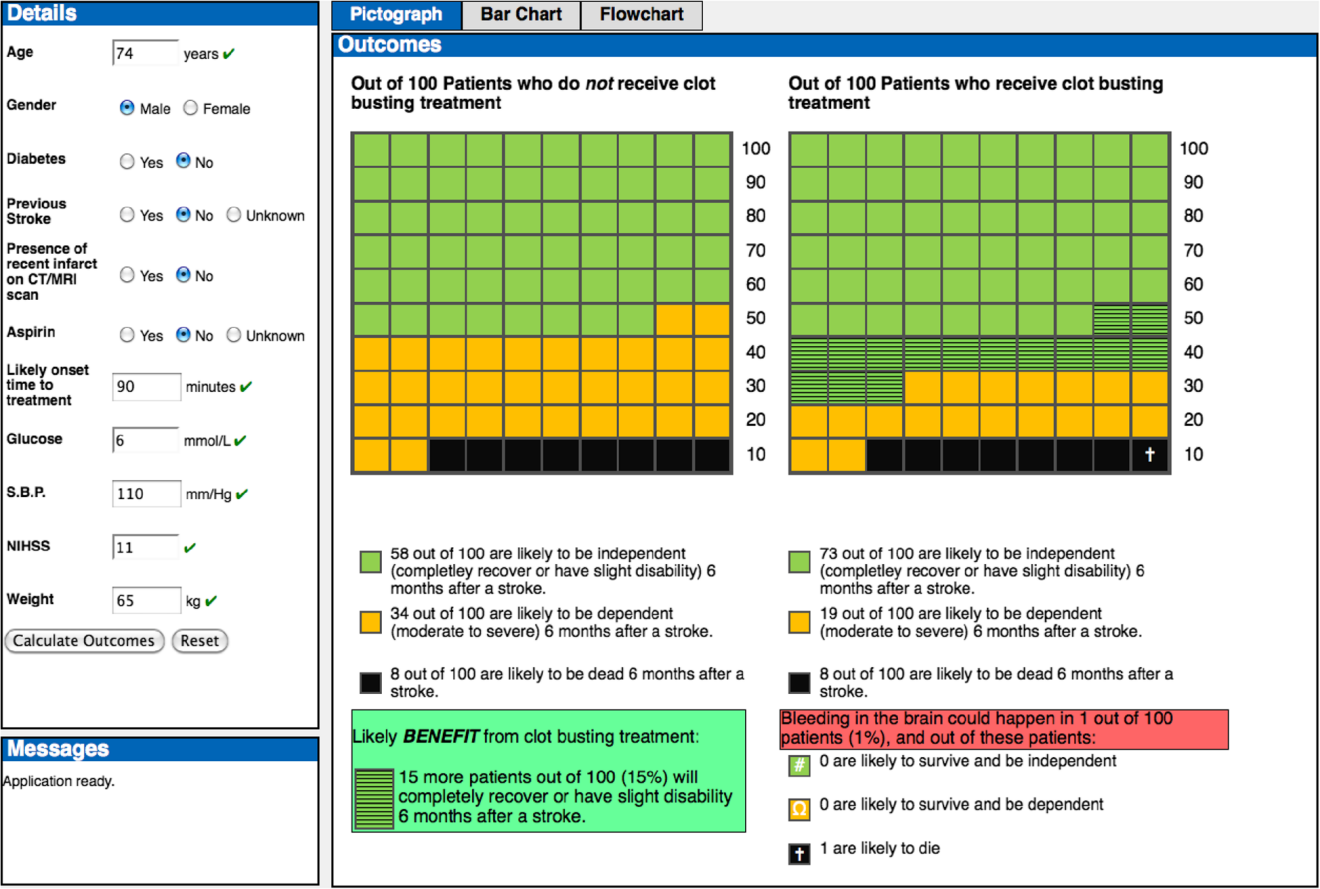
**Alpha prototype of COMPASS.**

The DAM was embedded within an alpha prototype of COMPASS, which was developed on an iPad® mobile digital device (Figure 2) for the following reasons: rapid input of patient information by clinicians; the large LCD touch sensitive screen facilitates accessibility and interpretation of the risk presentations by clinicians and patients/relatives; and for ease of deployment at the point of care without the need for additional peripherals or integration with existing hospital IT systems.

**Figure 3:**
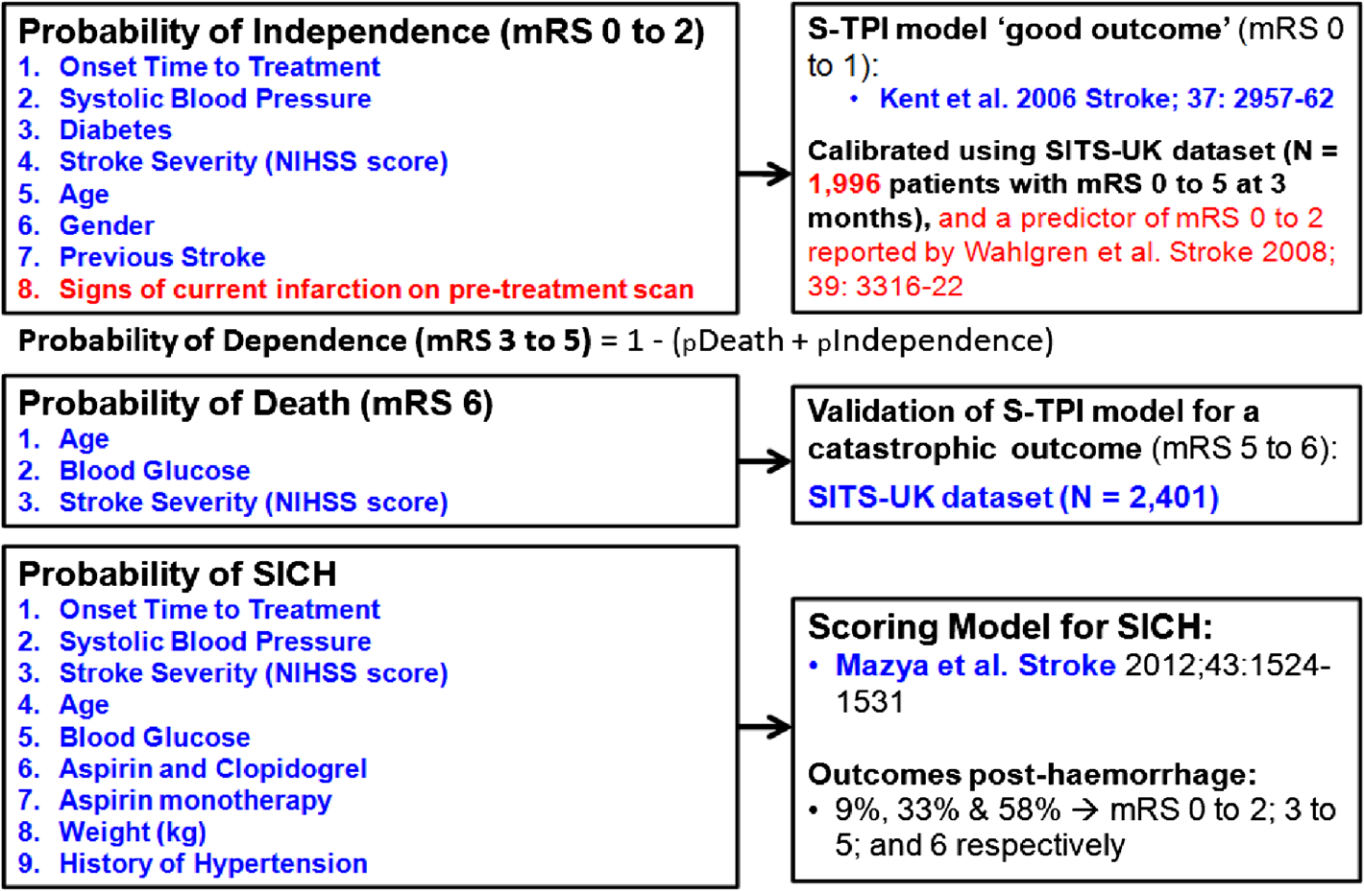
**Summary of Decision Analytic Model embedded within COMPASS.** Predictions for mRS 0 to 2, 3 to 5 and death in untreated patients were validated using data (N = 5,715) from untreated patients recorded in the Virtual International Stroke Trials Archive.

Numerical and graphical risk presentations based on participants’ preferences on their form and content (identified in interactive workshops) were embedded in the prototype to convey outcome probabilities for short-term acute stroke outcomes.

A series of user interface features were incorporated into the prototype, informed by design principles from human computer interaction [34]: (i) patient details and outcomes all displayed on one screen (without scrolling) to facilitate calculation/viewing of predicted clinical outcomes; (ii) instant updating of patient details when users changed one or more entered patient values to expedite re-calculation of outcomes; (iii) instant validation for continuous patient details in accordance with the licensing criteria for thrombolysis (green ticks and orange exclamation marks appear to the right of text boxes to indicate that entered continuous values are within or outwith the licensing criteria respectively, and red crosses to the right of text boxes to indicate that invalid values have been entered); and (iv) prompts and warning messages when entered values are invalid or outwith the licensing criteria for thrombolysis.

Populating the patient details (which would be undertaken by the treating clinician) and selecting ‘calculate outcomes’ generates outcome probabilities presented numerically (percentages and natural frequencies) and graphically (using pictographs, clustered bar graphs and a flowchart diagram juxtaposed with stacked bar graphs). Predicted net benefit and harm from thrombolysis (absolute difference between probability of independence with and without treatment) is presented in a summary box at the bottom left of the screen.

#### Usability testing phase

Informed by previous phases, we aimed (i) to test usability of an alpha prototype of COMPASS with clinicians and patients/relatives, in order to optimise the user interface and information content to enhance practicality, acceptability and usability in the actual acute stroke setting; and (ii) to establish the acceptability and feasibility of a beta prototype in the clinical setting based on experiences of clinicians and patients/relatives.

Interactive usability testing of the prototype was undertaken by 12 stroke clinicians (five stroke physicians, five emergency department physicians, two stroke nurse practitioners), plus five patients with a history of stroke and four of their relatives.

Usability testing utilised paper prototyping [35] to elicit clinicians’ preferences on screen appearance and layout (portrait [vertical] orientation with radio buttons for toggling between risk presentations; and two in landscape (horizontal) orientation with either radio buttons or tabs for toggling between risk presentations); chronological order of patient details; labels used to denote patient details; and content of risk presentations. Clinicians then used a functional prototype on the iPad, which was customised in accordance with each clinician’s preference on screen appearance/layout and content identified during paper-prototyping. Clinicians were encouraged to use COMPASS in a simulated way (e.g. entering data on hypothetical cases), and their comments and reactions during their interactions with the functional prototype were recorded by the two researchers (DF and DJN). The session ended with a brief interview about potential benefits/problems with use of COMPASS in clinical settings.

Patient/relative usability testing involved a demonstration of the risk presentations (paper and iPad screen showing two patient scenarios - one with clear and one with borderline benefit from treatment), followed by a brief interview to elicit their views and preferences on mode (paper or computerised presentation); type of risk presentation (e.g., pictograph); order, complexity and possible improvements that could be made to the risk presentations; and potential benefits/problems with use of the risk presentations during the hyper-acute period of stroke.

All data collected during usability testing were discussed with the research team in regular project meetings to inform production of a beta prototype of COMPASS and design of a subsequent feasibility study in the clinical setting.

#### Feasibility study

Over a six month period, 19 stroke physicians and stroke nurse practitioners (within three acute stroke units in England providing round the clock thrombolysis) were given access to COMPASS on iPad® mobile digital devices and a website. Each site was also supplied with a wireless printer. One of the authors (DF) provided clinicians with a face-to-face tutorial on use of COMPASS. A video tutorial on the fundamental operations of COMPASS was embedded within the iPad.

Clinicians used COMPASS pragmatically (i.e. at the discretion of the treating clinician; this approach to use of COMPASS was informed by discussions with clinical teams prior to the feasibility study) within their acute stroke pathway to support clinical decision-making for thrombolysis, and/or communication of the risks/benefits of treatment to patients/relatives. Paper-based self-completion forms (Additional file 2), interviews and computerised data logging (iPad) captured information on the use of COMPASS by clinicians. Interviews with patients/relatives explored their experiences of discussions about thrombolysis supported by COMPASS. Interviews were audio recorded and transcribed verbatim for the purposes of analysis.

Interviews with clinicians and patients/relatives were conducted by one researcher (DF), and followed a topic guide (see below). Interviews with clinicians took place in private offices within acute stroke units as soon as practicable following use of COMPASS. All interviews with patients/relatives who agreed to participate in an interview all took place in their homes within (~7+/−*2* days) after the stroke/thrombolysis decision making discussion supported by COMPASS.

### Interview guides used in the feasibility study

**A. Clinician Interviews**

***General issues connected with their experience of the consultation using the decision aid***

- As an introductory question - What is the present situation like (eligibility assessment and risk communication) without the decision aid?
- How did eligibility selection/consultations using the decision aid compare to a conventional eligibility assessment/consultation?

***Use of the decision aid for eligibility selection***

- Did you use the decision aid for eligibility selection?
- Did the outcomes generated by the decision aid help you make eligibility decisions?
- What are the benefits of using the decision aid for eligibility selection?
- What are the problems with using the decision aid for eligibility selection?
- If you did not use the decision aid for eligibility selection-could you please explain why?

***Role of the risk presentation tools***

- Did you use the decision aid for risk communication?
- What are the benefits of using the decision aid for risk communication?
- What are the problems with using the decision aid for risk communication?
- What risk presentations and strategies did you use?
- What information did you feel that you managed to convey to patients/relatives using the decision aid?
- How did patients/family members react to the risk presentations?
- If you did not use the decision aid for risk communication-could you explain why?

***Acceptability of the decision aid and data collection methods***

- In your view what are the barriers (and facilitators) to the use of the decision aid and its integration within the current care pathway for thrombolytic treatment in acute stroke?
- How could the support website and decision aid be improved?
- How could the methods of data collection be improved?

**B. Patient and Relative Interviews**

***General issues connected with their experience of the consultation***

- What information were you given about thrombolysis (clot-busting treatment) for stroke?
- Were you involved in the decision to have clot-busting treatment?
- How did you feel about being involved in the decision about clot-busting treatment?
- What things did you take into account when making your decision?

***Role of the risk communication tools:***

- How did the doctor/nurse explain the benefits/risks of clot-busting treatment to you?
- Were you shown risk and benefit information using pictures?
  - o If yes, could you tell us about this? (elicit information on mode [paper or on iPAD screen] and form (e.g., pictograph)
- Did the information on risks/benefits of clot-busting treatment help you to understand certain things? What? How?
- Was there too much information? Was there anything that was *not* clear?
- Did the information on risks/benefits help you make a decision? If yes, how?
- Would you have liked a copy of the information on benefits/risks of clot-busting treatment to keep? If yes why?
- What other information/support would have been helpful to you?

Interview data were subjected to an iterative conceptual content analysis [36] by one member of the research team (DF). A priori [based on topic guides] and emergent coding were used to summarise key themes for discussion with the research team who served as a challenge forum on the integrity of the analysis. Quotations from participants were used to represent key themes, and to enable the reader to adjudicate on the robustness of the interpretations. An integrative analysis of all data collected on use of COMPASS (paper-based self-completion forms, interviews and computerised data logging) were considered alongside our previous development work and relevant literature to inform production of a gamma prototype.

## Results

### Decision-analytic model

A decision-analytic model (DAM) was constructed to predict the patient-specific probability of acute stroke outcomes at three months, with and without thrombolysis, including risk of SICH and subsequent impact of SICH (Figure 3). The DAM also includes patient-specific predictions of risk of SICH for patients treated with thrombolysis (using a scoring model derived from patients treated with thrombolysis in routine practice [31]), including the subsequent impact of SICH on outcomes at three months with reference to proportions of patients that would be mRS 0 to 2, 3 to 5 and dead following SICH [32].

### Interactive group workshops

Clinicians stated that paper-based decision support was ‘unwieldy’ and computerised methods were likely to be the most efficient mode of delivery within the hyper-acute period of stroke. Computerised methods were considered the most efficacious mode of delivering decision support, and the draft risk presentations were considered useful for conveying short-term outcome probabilities to patients/relatives.

Presentation of short-term outcomes in patient/relative friendly-language (e.g., “clot-busting treatment” for thrombolysis) within verbal presentations by trusted clinicians, supported by using pictographs or clustered bar graphs (showing outcomes with and without thrombolytic treatment - expressed as percentages and natural frequencies with ‘out of 100 patients’ as the denominator) were identified as feasible methods for conveying a balanced presentation of the benefits and risks of thrombolytic treatment to patients/relatives. In contrast, long-term outcomes (e.g., life expectancy) elicited strong negative reactions from patients/relatives (i.e., highly likely to elicit fear).

### Usability testing

Clinicians reported potential benefits in enhanced decision-making about thrombolysis for individual patients within the licensing criteria, including better risk communication and informed consent. Clinicians expressed a clear preference for pictographs as a risk presentation/communication tool.

Potential perceived barriers to use were: clinicians’ acceptance of the outcome probabilities; capability of patients/relatives to understand the risk presentations; conveying an artificial level of certainty leading to potential problems with providing individualised information to patients/relatives; and the potential to interrupt clinical flow, and ultimately delay decision-making and treatment.

The language used to describe the options (treatment with and without thrombolysis) and outcome states (independence, dependence, death and SICH) conveyed in the risk presentations were comprehensible to patients/relatives. Patients/relatives revealed mixed preferences for paper-based or computerised risk presentations. A greater degree of involvement in the decision-making process and increased reassurance about a decision to consent to treatment, both before and after treatment (if they were provided with a copy of the risk presentations) were mentioned as benefits of the risk presentations. It was evident from comments made by patients/relatives that the risk presentations facilitated an understanding (i) of the more immediate risk of SICH associated with thrombolytic treatment and outcomes following SICH; (ii) of the absolute increase in functional independence (referred to colloquially as ‘hope’ or ‘life’) associated with treatment; and (iii) that overall mortality was equivalent with and without thrombolysis.

A majority considered it important to present a balanced synopsis of the risks and benefits of treatment, although there were mixed views on the value of conveying risk of SICH (especially when this was ‘small’; 1 in 100 patients). Several expressed a preference on outcomes presented in the order of independence, dependence and death. Concerns were raised by one patient and relative that the risk presentations may convey too much information during a highly stressful period (particularly the flowchart diagram), and emphasised that a focus could be placed on the summary box showing the likely net benefit from thrombolysis.

**Figure 4:**
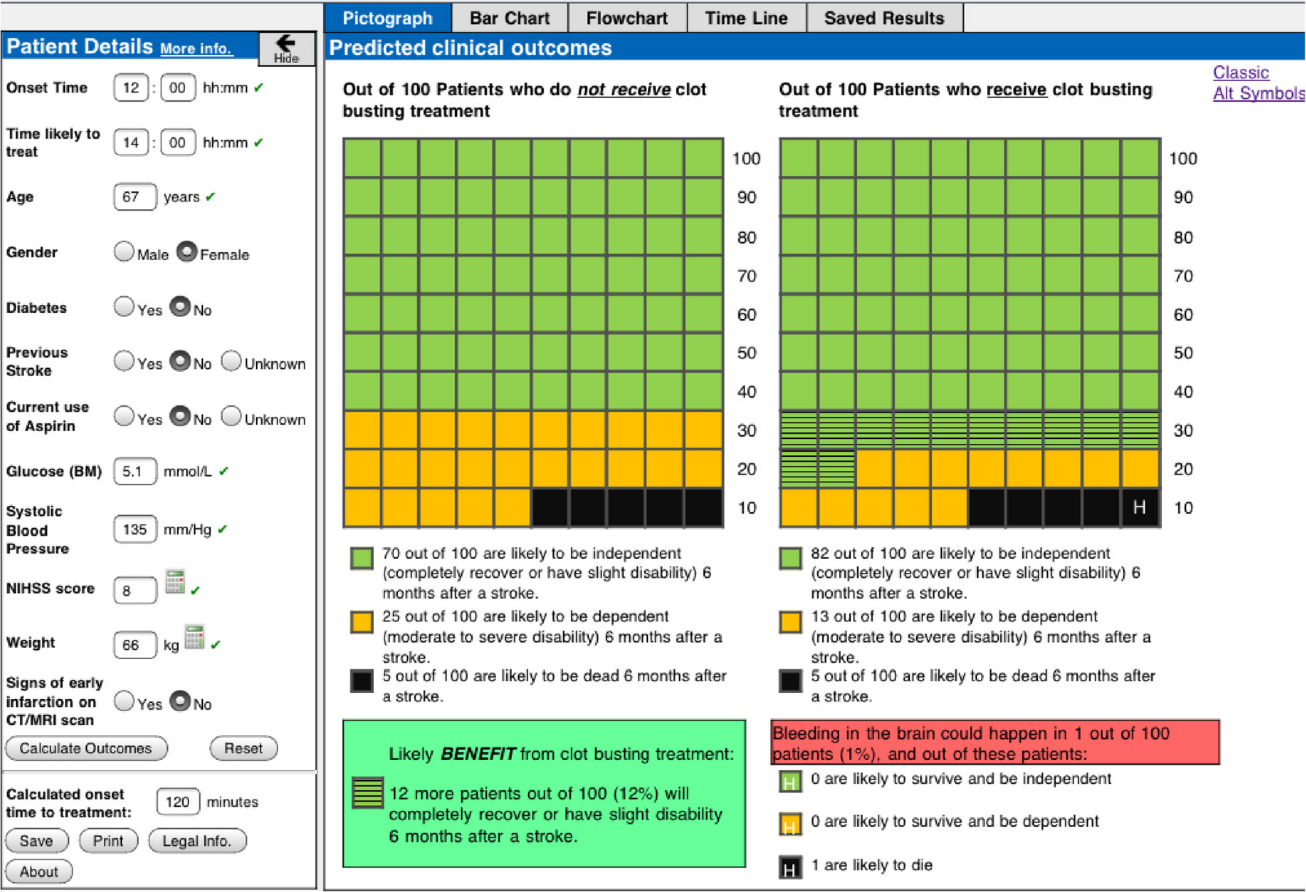
**Beta version of COMPASS.**

Usability testing informed amendments to the user interface, graphical risk presentations and inclusion of additional features (Table 1) to produce a beta prototype (Figure 4).

**Table 1:** **Amendments to alpha prototype of COMPASS resulting from usability testing**

**Amendment**	**Rationale**
• Landscape orientation with ‘tabs’ to switch between risk presentations	• Analogous to existing systems (e.g., Internet explorer)
• Headers ‘inputs’ and ‘outcomes’ amended to ‘Patient details’ and ‘Predicted clinical outcomes’ respectively	• Reflects the language used in clinical practice, and to reduce perception of an artificial level of certainty
• Order of patient details (demographics, medical history, blood results, examinations and CT scan)	• Sequence that information ‘typically’ becomes available during the hyperacute period
• Amendments to labels for patient details and menu of operational definitions for patient details	• Avoid ambiguity, expedite data entry and security with data validation
• Separate text boxes for entering information on stroke onset time and time likely to treat	• Security with data validation - with only one text box for ‘stroke onset time to treatment’ there is no reference point for stroke onset time or an explicit target treatment time
• Automatic deletion of entered values when editing (and clearing risk presentation to indicate that calculation of outcomes needs to be repeated)	• Security with data validation by reducing risk of data mis-entry/accidental changes to patient details
• Amendments to risk presentations:	• Consistency with preferences of clinicians and patients/relatives
o use of the letter H to denote SICH and impact of SICH in the pictograph for treated outcomes	
o re-ordering information in the clustered bar graph and flowchart diagram (independence, dependence, death)	
• Inclusion of additional features:	• Increased acceptability and usability - enhanced governance/consent processes; and facilitating case review and use as a clinical training aid
o weight conversion tool (Stones/lbs to kg);	
o NIHSS calculator;	
o ‘timeline’ function showing decrease in likely benefit from treatment as a function of stroke onset time to treatment;	
o ability to save and print the risk presentations

### Feasibility study

Data collected on contact forms and automatically logged data on use of COMPASS by clinicians are summarised in Table 2. Ten (out of 19 given access) clinicians reported using COMPASS for 25 patients (17 treated and eight not treated with thrombolysis) via the iPad (n = 23) or the web (n = 3) over the six month study period. COMPASS was used with 15 patients to support clinical decision-making or to obtain more detail on likely patient benefit after a decision to offer thrombolysis. Risk presentations generated by COMPASS were shared with 14 patients/relatives (predominately with relatives [n = 10] via the iPad screen [n = 11] using pictographs [n = 14]). In three cases this was before treatment to support informed consent, and in ten to augment understanding of the decision made about thrombolysis after treatment. Pictographs were used to facilitate understanding of a decision not to offer thrombolysis to one relative. One stroke physician used COMPASS as a clinical training aid with an emergency medicine physician to show the likely outcomes if a patient had arrived within the time window for thrombolysis. COMPASS was also used to assess the potential (missed) outcomes for a patient that had not been referred to the stroke team. Opportunities to use COMPASS, but where it was not used by clinicians were reported on eight occasions.

**Table 2:** **Data on use of COMPASS in the clinical setting**

**Generic pattern of use by clinicians (N = 10)**	**F (%F)**
**Cases**	
Treated patients	17
Untreated patients	8
Overall	25
**Platform**	
iPad	23
web	3
**Category of use**	
Clinical decision making	12
Obtain more detail on likely patient benefit	3
Risk presentations shared with relatives/patients	14
Other clinical activity	2
**Opportunity for use, but not used**	
Decision aid was unavailable	1
Not used for other reason	7
**Risk presentations shared with patients/relatives (N = 14)**	
**Period when risk presentation was shared**	
Before infusion	3
After infusion	10
Justify decision not to offer thrombolysis	1
**Risk presentation shared with:**	
Patient	1
Relative(s)	10
Patient and relative(s)	3
**Mode of risk presentation**	
iPad	11
Paper	3
**Form of risk presentation**	
Pictograph	14
Clustered bar chart	1
Flowchart/stacked bar graph	0
**Logged data, N = 21 cases**	
**Risk presentations viewed**	
Pictograph	21
Clustered Bar Graph	9
Flowchart and stacked bar graphs	6
**Use of additional features**	
NIHSS calculator	6
Weight convertor	6
Save function	6
Timeline	5
Print function	3
**Time in use (minutes)**	2.8 (7.6)*

*Median (IQR).

No adverse effects of use of COMPASS were reported.

The National Institutes of Health Stroke Scale (NIHSS) calculator (quantitative assessment of stroke-related neurologic deficit [37]), weight convertor tool and save function were each used for six cases. For five cases the timeline (showing decrease in net benefit from thrombolysis as a function of increasing stroke onset time to treatment) was used. The print function was used infrequently (n = 3). On three occasions data entry errors were detected by COMPASS and error messages given.

Time in use (first data input to calculation of outcomes following result of brain imaging to populate the data field ‘signs of current infarction on CT scan’) ranged from 0.7 to 30 minutes; the median (IQR) was 2.8 minutes (7.6 minutes).

Clinicians reported benefits in clinical decision-making: e.g. “*clear presentation of the risks and benefits*…..*able to look at the charts and say yes we should do this or* … *confirming your no*’ (Stroke Physician [SP] 2), especially for patients at extremes of the licensing criteria; e.g. the lower end of the NIHSS: “*confirmation that this low level of NIHSS had benefit*” (SP 6).

Benefits in risk communication were emphasised, in particular visual presentation of data:

*“feel comfortable saying actually five more people would benefit, there’s no change in risk of death”* (Nurse Practitioner 1).

“*there’s no significant additional mortality to the natural history’ …. that’s very, very difficult information to communicate without that sort of pictogram” *(SP 4).

One clinician emphasised the value of graphical risk presentations to support provision of post-decision information to relatives who were not present at the time of treatment: “*useful to tell the family and then explain what that treatment was and why it was or wasn’t a clear decision”* (SP 6).

Improved support to clinical governance and medico-legal issues were highlighted as benefits of COMPASS*:*

*“emphasises the importance of not only documenting a very high quality conversation but also puts our focus of mind that this is an important piece of managing the patient in that very difficult time”* (SP 3).

“*it then becomes part of the record which I think will stand up better in court”* (SP 4).

One clinician encountered difficulties with use of the bar graph. Nevertheless, clinicians generally considered that relatives found the risk presentations [pictographs] beneficial for risk communication and enhancing engagement: “*They get more engaged rather than just dazed when we explain the benefit and risk and they get to see something and they’re more focused on what we’re discussing*” (SP 3).

Seven themes on barriers to use of COMPASS were identified from interviews with clinicians and data from self-report forms: (i) when stroke physicians were involved in remote consultations with emergency medicine physicians; (ii) iPad not charged/unavailable for use; (iii) complex cases involving a consideration of variables not listed in COMPASS; (iv) inexperience with using computer technology/iPad; (v) confidence in accepting data on outcomes for patients at the extremes of the licensing criteria; (vi) patients clearly within the licensing criteria for treatment; and (vii) clinicians’ reservations about sharing information on ‘large’ probabilities of death/poor outcomes with patients/relatives.

Interviews with patients (n = 2) and relatives (n = 6) described how features of the graphical risk presentations (juxtaposition of displays with and without treatment and use of colour) enhanced their comprehension of the risks/benefits of treatment, including increased comfort with providing consent for thrombolysis and involvement in decision-making:

*“especially in such stressful circumstances, someone just quoting figures at you one in this and two in that……..you can compare the pictures alongside each other rather than somebody saying you know well 20% this and 25% that”* (Relative 4).

*“It gave me as you say a visual sort of explanation of it which I couldn’t have taken in mentally, not at that time”* (Relative 2).

*“green’s for go you know…..to me, is a positive thing”* (Relative 3).

One relative would have preferred one-to-one verbal presentation only and another would have preferred not to have received information on death: *“I don’t think that the information about one in however many within a year dies……. I thought ‘I don’t really need that information at the minute”* (Relative 4)

The value of being given a paper copy of the risk presentations to keep was noted by one relative (it enabled reflection on the consent discussion and provided reassurance that the most appropriate decision had been made): “*I was able to just reflect and say okay I’ve done the right thing for my wife”* (Relative 3)

**Figure 5:**
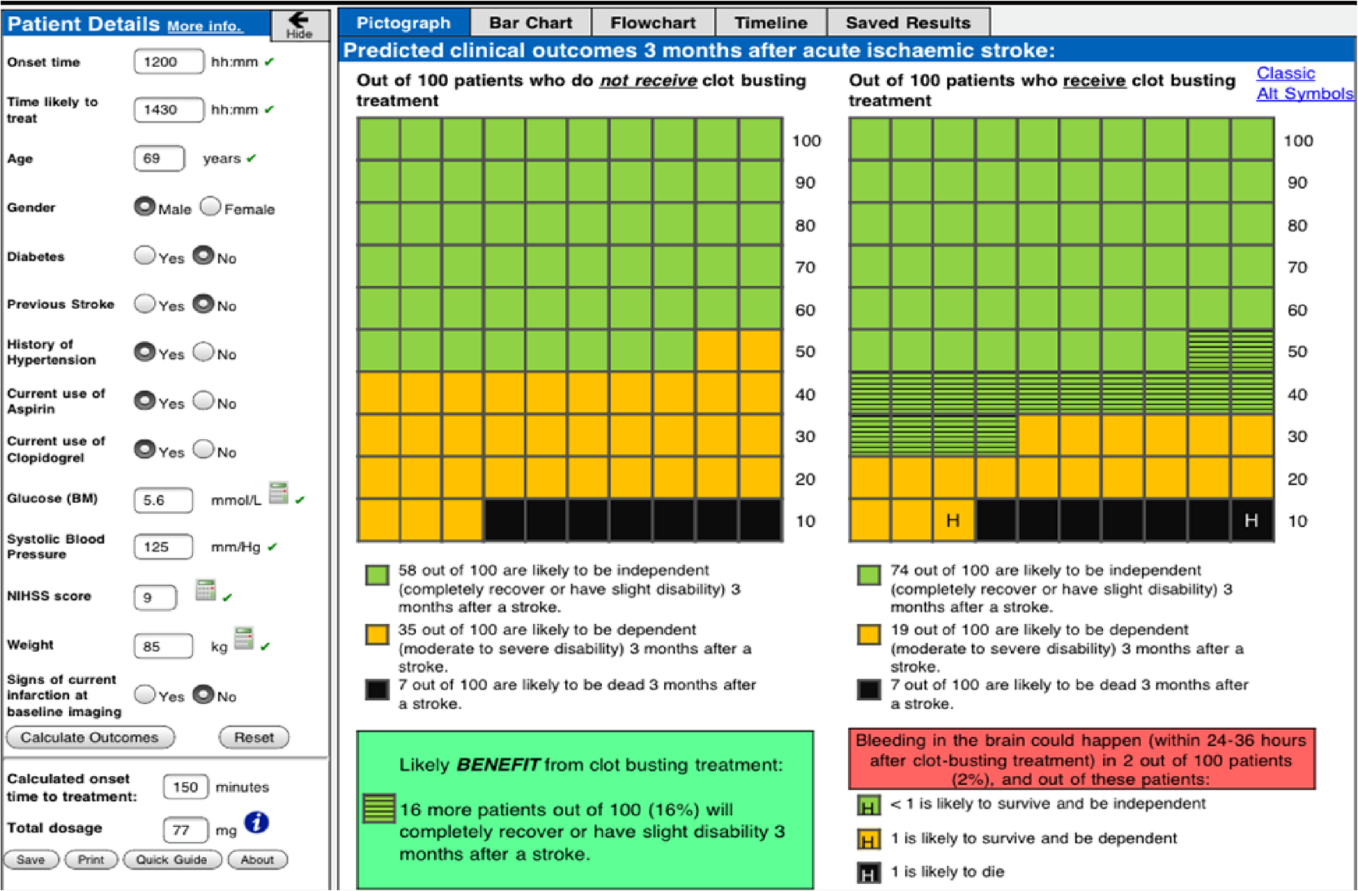
**Gamma version of COMPASS.**

Findings and subsequent discussions within the research team informed amendments to COMPASS (Table 3) to produce a gamma prototype (Figure 5). Details of the full range of additional features in the gamma prototype are shown in Additional file 3.

**Table 3:** **Amendments to the beta version of COMPASS following feasibility testing in the clinical setting**

**Amendment**	**Rationale**
**Revisions to decision analytic model**	• Enhanced clinical face validity of predicted outcomes and accuracy of predicted risk of SICH
o Time horizon of three months for predicted outcomes (independence, dependence and death)	
o Inclusion of new scoring model for risk of symptomatic intracranial haemorrhage, which necessitated the addition ‘current use of Clopidogrel’ and ‘history of hypertension’ to the list of patient details	
I**nclusion of the additional features:**	• Enhanced clinical utility and interpretability
o rt-PA dosage calculator, with a pop-up icon displaying detailed dosage figures	
o Glucose conversion tool (mg/dl to mmol/L)	
o Line graph incorporated into timeline function to show more clearly the decrease in likely benefit from thrombolysis as a function of stroke onset time to treatment	
**Amendments to list of patient details and warning messages:**	• Enhanced clinical face validity and usability, reduced risk of data entry errors and security with data validation
o Amended warning for entered NIHSS values < 5 and > 25: “The license states that a minor neurological deficit or severe stroke as assessed clinically (NIHSS > 25) are relative contraindications to treatment with rt-PA. For patients with mild stroke the risks may outweigh the expected benefit. Patients with very severe stroke are at increased risk of intra-cerebral haemorrhage.”	
o Rules for number of integers that need to be entered for onset time, target treatment time, age, systolic blood pressure, glucose and weight); e.g. users must enter >1 and <4 integers for systolic blood pressure	
o Signs of early infarction on CT/MRI scan replaced with ‘Signs of current infarction at baseline imaging’	
o Larger text boxes for two patient details: systolic blood pressure and glucose (BM)	
o Added flexibility for stoke onset time and target treatment time – users can enter values in multiple formats (hhmm, hh:mm, hh.mm)	
**Amendments to the risk presentations:**	• Enhanced interpretability of predicted clinical outcomes
o Addition of time horizon for SICH ‘within 24–36 hours after clot-busting treatment’	
o Added to whitespace area: “Please note: predicted clinical outcomes at 3 months apply to patients with pre-stroke modified Rankin scores of 0 to 2”	
**Other amendments to the user interface:**	• Enhanced usability and
o Inclusion of ‘acute ischaemic stroke’ to header ‘predicted clinical outcomes’	• acceptance of the decision aid
o Disabled copy/paste function (tablet computer only)	
o Inclusion of readability statistics and production date

## Discussion

This is the first study to develop and pilot test the use of a decision aid for treatment of acute stoke with intravenous thrombolysis in the clinical setting. COMPASS has been designed in an effort to support: (i) the clinical decision to offer thrombolysis based on individual differential effectiveness, (ii) clinicians with a mechanism to rapidly communicate the probability of a good clinical outcome and the risks of thrombolysis with patients/relatives in order to respect their autonomy; and (iii) clinicians to assess the degree to which patients/relatives desire to engage in thrombolysis decision-making prior to making the decision to administer treatment.

The findings of the feasibility study provides evidence that COMPASS may have tangible benefits in the clinical setting for supporting patient-specific eligibility selection for thrombolysis in the treatment of acute ischaemic stroke and personalised risk communication, including support for recording of decision-making. The decision aid also has potential use as a clinical training aid. COMPASS supports ‘instant validation’ of entered patient values on continuous variables in accordance with the current licensing criteria for thrombolysis. Various scenarios (based on real or simulated patients) can be used to facilitate learning about assessment of eligibility for thrombolysis, including absolute and relative contradictions for treatment within the current licensing criteria and likely clinical outcomes at three months after stroke. The graphical risk presentations can also be used to develop skills in communicating benefits and risks to patients and their relatives in the acute setting. Furthermore, additional features such as the NIHSS and dosage calculators can be used to facilitate training on assessment of stroke severity and total rt-PA dose (mg), bolus (ml), IV infusion (ml/hr) and number of 50 mg rt-PA vials needed. Finally, the extreme time dependency of treatment and the need for expeditious door to needle times can be modelled by using the timeline function.

The benefits of involving patients/relatives and clinicians in an iterative co-design and development process (with reference to evidence-based methods to present a balanced synopsis of probabilistic information on benefits/risks) ensures that the mode, form and information content of COMPASS is responsive to users’ preferences and the complexities of the decision context [6,33]. Furthermore, it enabled the development of a different type of decision aid to those used in non-emergency settings, and which addressed shortcomings of currently available tools for supporting decision-making and patient understanding in the treatment of acute stroke with thrombolysis [6].

The ability of COMPASS to rapidly present individualised outcome probabilities has potential benefits over aggregate-level estimates to support eligibility decision-making in two ways: (i) enhanced comfort/confidence with thrombolysis decisions, in particular for patients at the extremes of the licensing criteria; and (ii) minimising ‘black and white’ decision-making (based exclusively on whether or not a patient is within the licensing criteria) by emphasising a need to consider the magnitude of likely net benefit/risk for any individual patient. This represents more effective and appropriate patient selection in comparison to target driven or binary decision-making based on licensing criteria alone.

Time in use was within acceptable parameters. The outlying value of 30 minutes represents inputting of initial demographic data then entering once CT scan had been confirmed (as opposed to waiting for CT scan to be confirmed before populating all data fields). Any negative impact on clinical flow/door to needle time (arrival at hospital to administration of treatment) which may delay treatment decision-making and thrombolysis (due to additional time needed to explain the content of risk presentations to patients and relatives) can be minimised by using COMPASS in parallel to other processes along the thrombolysis pathway so that delay is minimised e.g. whilst waiting for brain imaging.

Use of COMPASS in the feasibility study after treatment might suggest a primary use to justify decisions in accordance with a paternalistic model of decision-making. However, the majority of cases had clear net benefit and clinicians reported enhanced communication with patients/relatives, including conveying risk of SICH which patients may find difficult to process [20]. The latter is important, as acute stroke is often experienced by patients/relatives as a traumatic event, which can impede their capacity to understand verbal information conveyed by clinicians [3].

Comprehension of potential benefit versus harm of treatment, including increased comfort with providing consent for thrombolysis and engagement in decision-making were identified as possible benefits of the risk presentations with patients/relatives. The use of pictographs to convey probabilistic information is consistent with research reporting on their acceptability in people with differing health literacy skills, including facilitating the acquisition of verbatim (specific probabilistic information) and gist knowledge (general impression) [38].

Issues related to clinicians’ acceptance of probabilities highlights situations where engaging patients/relatives (where appropriate) in shared decision making with clinicians may be the most appropriate approach. Outcomes generated by COMPASS represent choice-based decisions under conditions of uncertainty involving trade-offs between the likely long-term benefit (reduced risk of significant post-stroke disability) and short-term risk of SICH and its consequences, which are likely to be valued differently by individual patients/relatives [20-22]. However, there are varying individual preferences for information on thrombolysis (e.g. for mortality identified in our study) and involvement in decision-making [20,21].

COMPASS affords potential for further strengthening relational decision support practices by providing an additional mechanism to help clinicians to guide patients/relatives through the thrombolysis decision-making process, including augmenting patient/relative autonomy by facilitating their active involvement in thrombolysis decision-making.

Generalisability of our results must be made cautiously due to the limited sample sizes of patients/relatives and clinicians in the feasibility study. Analysis of the interviews was also performed by a single author (DF), although any potential bias was minimised by engaging the other authors in the role of peer reviewers/debriefers (i.e., emerging themes were discussed within group meetings) to ensure the conceptual interpretations were a credible account of the participants’ experiences.

A prospective evaluation in other centres and health care systems (along with skills training for clinicians on risk communication), with larger samples of stroke clinicians and patients/relatives, to assess the utility and impact of the gamma prototype on thrombolysis rates, clinical outcomes, healthcare utilisation and safety, without compromising door-to-needle time is warranted. Further work is also needed to optimise use of COMPASS as a clinical training aid, and how it could be embedded/adapted for use within the telemedicine model of acute stroke care, including adoptability within other systems designed to facilitate rapid assessment of patient eligibility for thrombolysis.

Gamma prototype versions of COMPASS have been developed for smartphone, desktop and tablet computers to address issues related to accessibility. Relevance and quality of information content of COMPASS may diminish rapidly over time due to availability of new data on effectiveness of thrombolysis, including information systems designed to deliver decision support [6]. Therefore, to address these threats to ‘temporal validity’ there is a requirement to secure resources for supporting routine maintenance and updating of information content to support protracted use of decision aids such as COMPASS, including weighing up the pros and cons of implementation informed by prospective evaluation from randomised trials or real-time service evaluations [6,24,39].

## Conclusions

COMPASS may have tangible benefits in supporting patient-specific clinical decision-making about thrombolysis, and in risk communication with patients/relatives to augment understanding of thrombolysis and support with recording of thrombolysis decisions, including where appropriate increasing engagement of patients/relatives in shared decision making. Acceptability and functionality of COMPASS in other centres and health care systems (with larger samples of stroke clinicians and patients/relatives); including impact on door-to-needle times and thrombolysis rates requires prospective assessment in the clinical setting.

## Additional files

Additional file 1:
**Draft paper-based tools.** Examples of draft paper to support eligibility decision making about thrombolysis for individual patients (structured look-up tables and tables of decisions rules for different levels of net benefit from thrombolysis); and clinical communication of personalised information on the risks/benefits of thrombolysis to patients/relatives (clustered and stacked bar graphs, pictographs and flowchart diagrams). Additional file 2:
**Paper-based self-completion form.** Paper-based self-completion form used to collect data on use of COMPASS in the clinical setting by clinicians during the feasibility study. Additional file 3:
**Overview of Additional Features in COMPASS.** Additional features in the gamma prototype version of COMPASS.

## Abbreviations

COMPASS: COMPuterised decision Aid for Stroke thrombolySis
DAM: Decision-analytic model
NIHSS: National Institutes of Health Stroke Scale
mRS: Modified rankin scale
SICH: Symptomatic intracranial haemorrhage
SP: Stroke physician
S-TPI: Stroke-thrombolytic predictive instrument
