# Supplementary material for: Development of a computerised decision aid for thrombolysis in acute stroke care
Source: BMC Med Inform Decis Mak. 2015 Feb 7;15:6. doi: 10.1186/s12911-014-0127-1 (PMC4326413; doi:10.1186/s12911-014-0127-1)

Additional file 1: Draft paper-based tools

A: Structured look up table

NIHSS 13-18, Female, No History of Stroke, Age = 80

		Onset Time to Treatment (minutes)																		
		0	15	30	45	60	75	90	105	120	135	150	165	180	195	210	225	240	255	270
Systolic BP mm/Hg	180	0.31	0.31	0.31	0.31	0.31	0.31	0.31	0.31	0.31	0.31	0.31	0.31	0.31	0.31	0.31	0.31	0.31	0.31	0.31
		0.62	0.61	0.59	0.58	0.56	0.55	0.54	0.52	0.51	0.49	0.48	0.47	0.45	0.44	0.42	0.41	0.40	0.38	0.37
		**0.31**	**0.29**	**0.28**	**0.27**	**0.25**	**0.24**	**0.22**	**0.21**	**0.20**	**0.18**	**0.17**	**0.15**	**0.14**	**0.13**	**0.11**	**0.10**	**0.08**	**0.07**	**0.06**
	170	0.32	0.32	0.32	0.32	0.32	0.32	0.32	0.32	0.32	0.32	0.32	0.32	0.32	0.32	0.32	0.32	0.32	0.32	0.32
		0.66	0.64	0.63	0.62	0.60	0.59	0.58	0.56	0.55	0.54	0.52	0.51	0.49	0.48	0.47	0.45	0.44	0.42	0.41
		**0.33**	**0.32**	**0.31**	**0.29**	**0.28**	**0.27**	**0.25**	**0.24**	**0.23**	**0.21**	**0.20**	**0.18**	**0.17**	**0.16**	**0.14**	**0.13**	**0.11**	**0.10**	**0.09**
	160	0.33	0.33	0.33	0.33	0.33	0.33	0.33	0.33	0.33	0.33	0.33	0.33	0.33	0.33	0.33	0.33	0.33	0.33	0.33
		0.69	0.68	0.67	0.66	0.64	0.63	0.62	0.60	0.59	0.58	0.56	0.55	0.53	0.52	0.51	0.49	0.48	0.46	0.45
		**0.36**	**0.35**	**0.33**	**0.32**	**0.31**	**0.30**	**0.28**	**0.27**	**0.26**	**0.24**	**0.23**	**0.21**	**0.20**	**0.19**	**0.17**	**0.16**	**0.14**	**0.13**	**0.12**
	150	0.35	0.35	0.35	0.35	0.35	0.35	0.35	0.35	0.35	0.35	0.35	0.35	0.35	0.35	0.35	0.35	0.35	0.35	0.35
		0.73	0.72	0.70	0.69	0.68	0.67	0.66	0.64	0.63	0.62	0.60	0.59	0.58	0.56	0.55	0.53	0.52	0.51	0.49
		**0.38**	**0.37**	**0.36**	**0.35**	**0.34**	**0.32**	**0.31**	**0.30**	**0.28**	**0.27**	**0.26**	**0.24**	**0.23**	**0.22**	**0.20**	**0.19**	**0.17**	**0.16**	**0.15**
	140	0.36	0.36	0.36	0.36	0.36	0.36	0.36	0.36	0.36	0.36	0.36	0.36	0.36	0.36	0.36	0.36	0.36	0.36	0.36
		0.76	0.75	0.74	0.73	0.72	0.70	0.69	0.68	0.67	0.65	0.64	0.63	0.62	0.60	0.59	0.58	0.56	0.55	0.53
		**0.40**	**0.39**	**0.38**	**0.37**	**0.36**	**0.35**	**0.34**	**0.32**	**0.31**	**0.30**	**0.29**	**0.27**	**0.26**	**0.25**	**0.23**	**0.22**	**0.20**	**0.19**	**0.18**
	130	0.37	0.37	0.37	0.37	0.37	0.37	0.37	0.37	0.37	0.37	0.37	0.37	0.37	0.37	0.37	0.37	0.37	0.37	0.37
		0.79	0.78	0.77	0.76	0.75	0.74	0.73	0.71	0.70	0.69	0.68	0.67	0.65	0.64	0.63	0.61	0.60	0.59	0.57
		**0.42**	**0.41**	**0.40**	**0.39**	**0.38**	**0.37**	**0.36**	**0.35**	**0.34**	**0.32**	**0.31**	**0.30**	**0.29**	**0.27**	**0.26**	**0.25**	**0.23**	**0.22**	**0.21**
	120	0.38	0.38	0.38	0.38	0.38	0.38	0.38	0.38	0.38	0.38	0.38	0.38	0.38	0.38	0.38	0.38	0.38	0.38	0.38
		0.81	0.81	0.80	0.79	0.78	0.77	0.76	0.75	0.74	0.73	0.71	0.70	0.69	0.68	0.67	0.65	0.64	0.63	0.61
		**0.44**	**0.43**	**0.42**	**0.41**	**0.40**	**0.39**	**0.38**	**0.37**	**0.36**	**0.35**	**0.34**	**0.32**	**0.31**	**0.30**	**0.29**	**0.27**	**0.26**	**0.25**	**0.24**

At each interval, results are presented in the following order:
* No treatment

* Treatment with rt-PA

No change / survival LOSS in independent state from treatment

Survival GAIN in independent state from treatment

B: Tables of decision rules

NIHSS 13-18
Male, History of Stroke, Age 80

		OTT windows for absolute gains in QALYs			OTT windows for absolute gains in the proportion of survival - independent state (mRS 0-2) at 6 months			Proportion dead at 6 months
		> 0	≥ 0.25 (3 mths)	≥ 0.5 (6 mths)	> 0	≥ 5	≥ 10	
Systolic BP	180	≤ 15	**	**	**	**	**	9 out of 100 (treated)<br>9 out of 100 (not treated)
	170	≤ 45	**	**	≤ 45	**	**	
	160	≤ 75	**	**	≤ 75	≤ 30	**	
	150	≤ 105	**	**	≤ 105	≤ 60	**	
	140	≤ 135	**	**	≤ 135	≤ 90	≤ 30	
	130	≤ 165	≤ 15	**	≤ 165	≤ 120	≤ 60	
	120	≤ 195	≤ 60	**	≤ 195	≤ 150	≤ 90	

Consult relevant look-up tables for specific figures

		OTT windows for absolute gains in mean life expectancy			OTT windows for absolute gains in expected time - independent state (mRS 0-2)			OTT windows for absolute decreases in expected time - dependent state (mRS 3-5)			OTT windows for absolute gains in the proportion of survival at 5 years		
		> 0	≥ 0.25 (3 mths)	≥ 0.5 (6 mths)	> 0	≥ 0.25 (3 mths)	≥ 0.5 6 mths)	> 0	≥ 0.25 (3 mths)	≥ 0.5 (6 mths)	> 0	≥ 5	≥ 10
Systolic BP	180	≤ 15	**	**	≤ 15	**	**	**	**	**	**	**	**
	170	≤ 45	**	**	≤ 45	**	**	≤ 45	**	**	≤ 15	**	**
	160	≤ 75	**	**	≤ 75	**	**	≤ 75	**	**	≤ 45	**	**
	150	≤ 105	**	**	≤ 105	≤ 15	**	≤ 105	**	**	≤ 75	**	**
	140	≤ 135	**	**	≤ 135	≤ 45	**	≤ 135	**	**	≤ 105	**	**
	130	≤ 165	**	**	≤ 165	≤ 75	**	≤ 165	**	**	≤ 135	**	**
	120	≤ 195	≤ 15	**	≤ 195	≤ 105	**	≤ 195	**	**	≤ 165	**	**

** At this systolic BP value there is not an OTT window associated with a treatment benefit for this category

C: Flowchart diagram - horizontal orientation

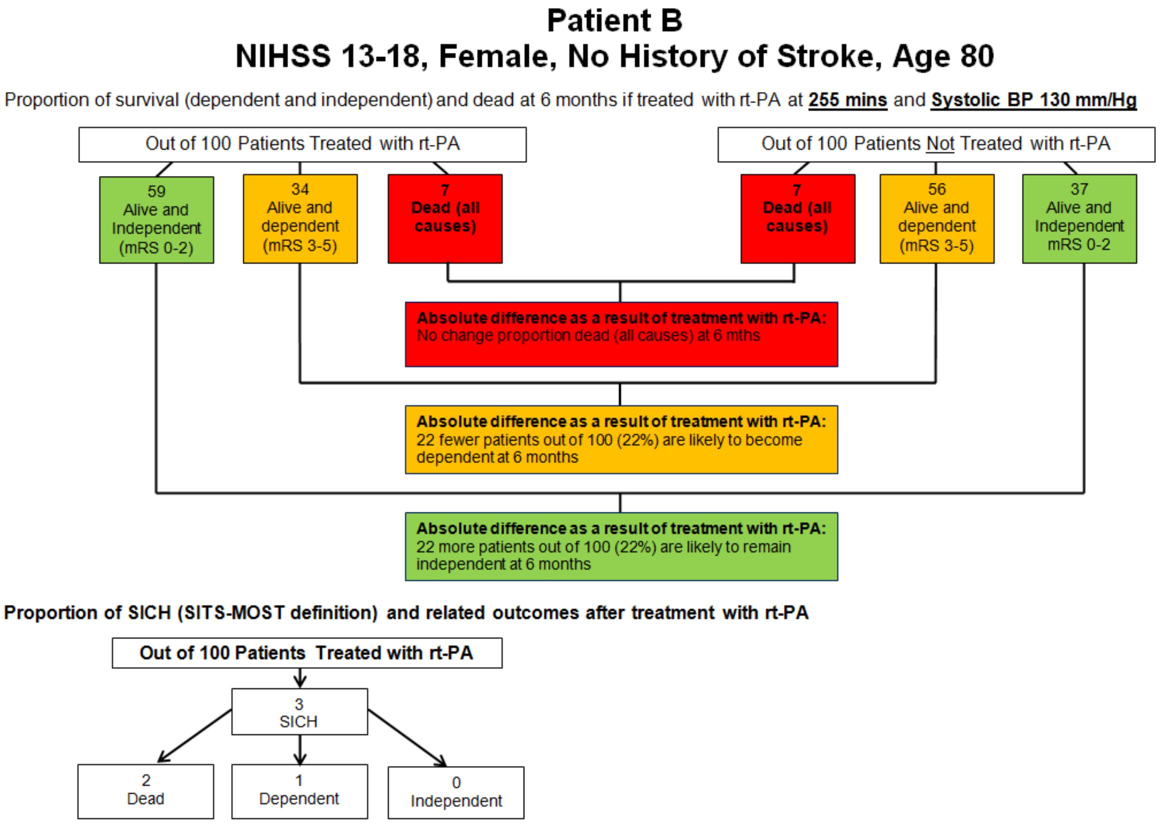

D: Pictograph

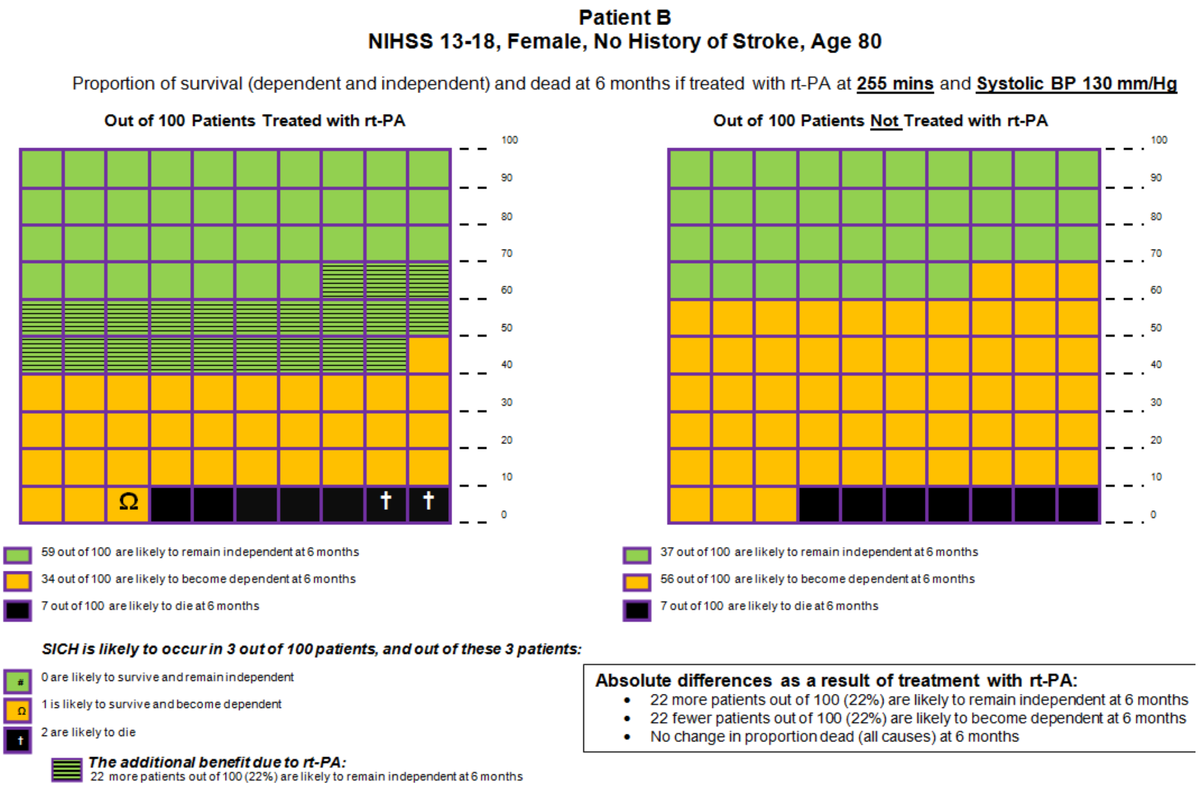

E: Stacked bar graph (independence, dependence and death)

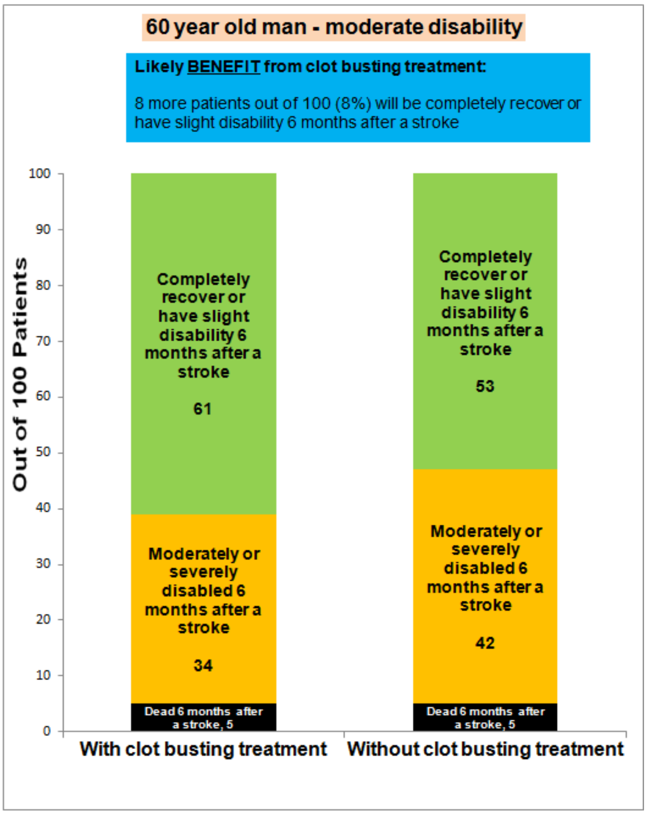

F: Clustered bar graph (life expectancy and expected time in independent and dependent state)

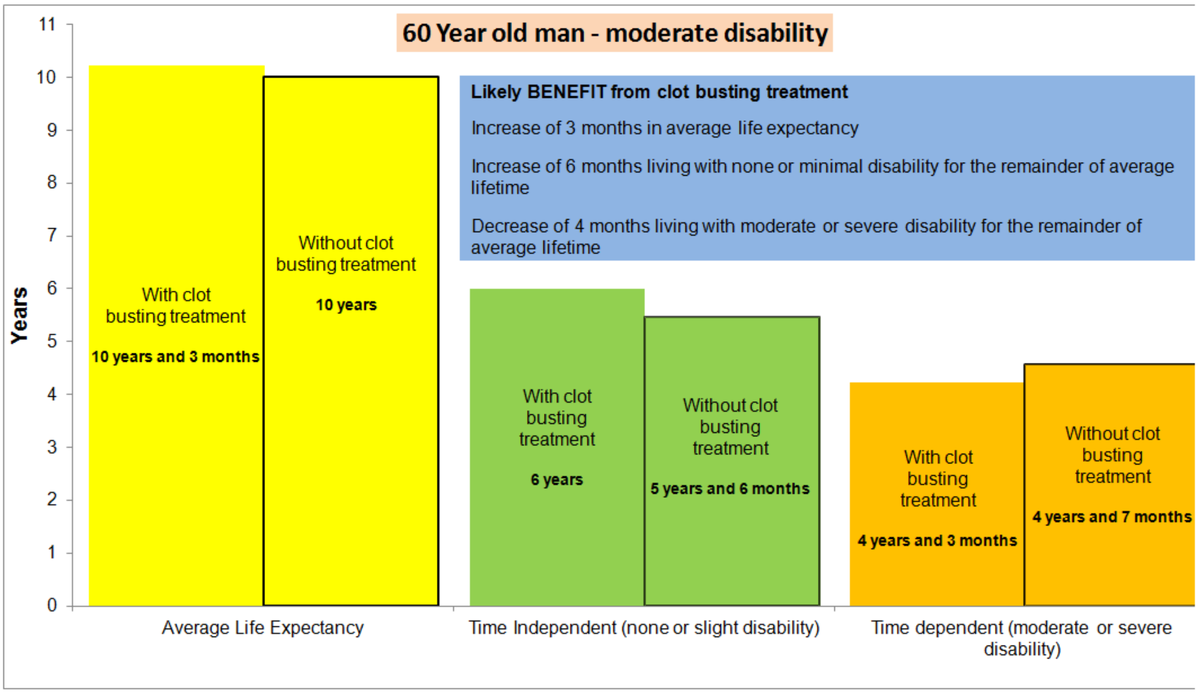

Supplement: Additional file 1: — Draft paper-based tools. Examples of draft paper to support eligibility decision making about thrombolysis for individual patients (structured look-up tables and tables of decisions rules for different levels of net benefit from thrombolysis); and clinical communication of personalised information on the risks/benefits of thrombolysis to patients/relatives (clustered and stacked bar graphs, pictographs and flowchart diagrams). [file 12911_2014_127_MOESM1_ESM.pdf]
