# Supplementary material for: Development of a computerised decision aid for thrombolysis in acute stroke care
Source: BMC Med Inform Decis Mak. 2015 Feb 7;15:6. doi: 10.1186/s12911-014-0127-1 (PMC4326413; doi:10.1186/s12911-014-0127-1)
Supplement: Additional file 2: — Paper-based self-completion form. Paper-based self-completion form used to collect data on use of COMPASS in the clinical setting by clinicians during the feasibility study. [file 12911_2014_127_MOESM2_ESM.pdf]

Additional file 2. Paper-based self-completion form

**A** Was the patient treated with IV rt-PA?	**B** Was the decision aid used? ***(enter all that apply)***	**C** If NO for B, please could you briefly state why the decision aid was not used on this occasion?	**D** Platform used	**E** Time taken to use (approx)	**F** How was the decision aid useful for eligibility selection?	**G** Method of presenting information to patients / families? (***enter all that apply***)	**H***** Did the decision aid report any errors? If YES please state:	**I** Would you use the decision aid again?	**J** If you answered NO to I, please could you briefly explain why?
**Yes or No**	**Any of 1, 2, 3, 4 or 5**	**Free Text**	**1, 2, 3 or 4**	**in minutes**	**Free Text**	**Any of 1, 2, 3, 4, 5, 6 or N/A**	**Free Text**	**Yes or No**	**Free Text**

**B. Was the decision aid used?**
1 = yes for eligibility decision making
2 = yes (consent with patient)
3 = yes (consent with family)
4 = yes (consent with patient + family)
5 = no

**D. Platform used**
1 = iPAD
2 = Web
3 = Stand-alone version
4 = Smartphone

**G. Method of presenting information to patients / families?**
1 = verbal presentation only
2 = risk presentations (iPAD)
3 = risk presentations (paper)
4 = pictograph
5 = bar graph
6 = flowchart
7 = N/A

Any further feedback would be greatly appreciated. You can leave further feedback on the DASH II website and get in touch with the DASH II team by email
