# Supplementary material for: Development of a computerised decision aid for thrombolysis in acute stroke care
Source: BMC Med Inform Decis Mak. 2015 Feb 7;15:6. doi: 10.1186/s12911-014-0127-1 (PMC4326413; doi:10.1186/s12911-014-0127-1)
Supplement: Additional file 3: — Overview of Additional Features in COMPASS. Additional features in the gamma prototype version of COMPASS. [file 12911_2014_127_MOESM3_ESM.pdf]

Additional File 3. OVERVIEW OF ADDITIONAL FEATURES OF COMPASS

Calculator icons to the right of several text boxes can be used to access additional features, which in current practice are largely undertaken using paper-based materials.

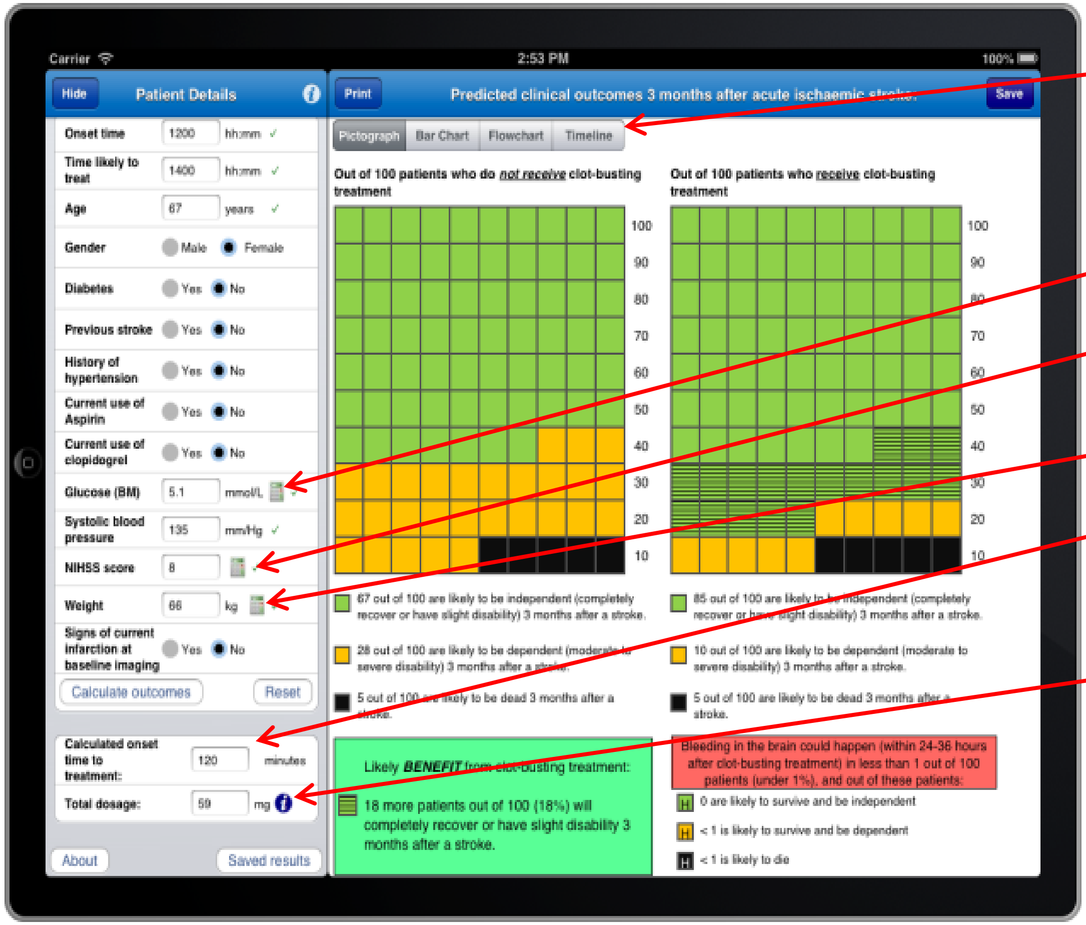

- **Timeline graph** – selecting this tab shows the decrease in predicted net benefit from thrombolytic treatment as a function of increasing stroke onset time to treatment (see next page)
- **Glucose convertor (mg/dl to mmol/L)**
- **National Institute of Health Stroke Scale** (NIHSS) – see next page
- **Weight conversion** (stones/lbs to Kg)
- **Calculated onset time to treatment in minutes** (derived from patient values entered for onset tome and estimated time likely to treat)
- **Thrombolysis (rt-PA) dosage calculator** (derived from value entered for a patient's weight).

  Selecting the shows figures for:
  - Total rt-PA dose (mg)
  - 10% bolus (ml)
  - 90% IV infusion (ml/hr)
  - No of 50mg rt-~PA vials needed

NIHSS Calculator

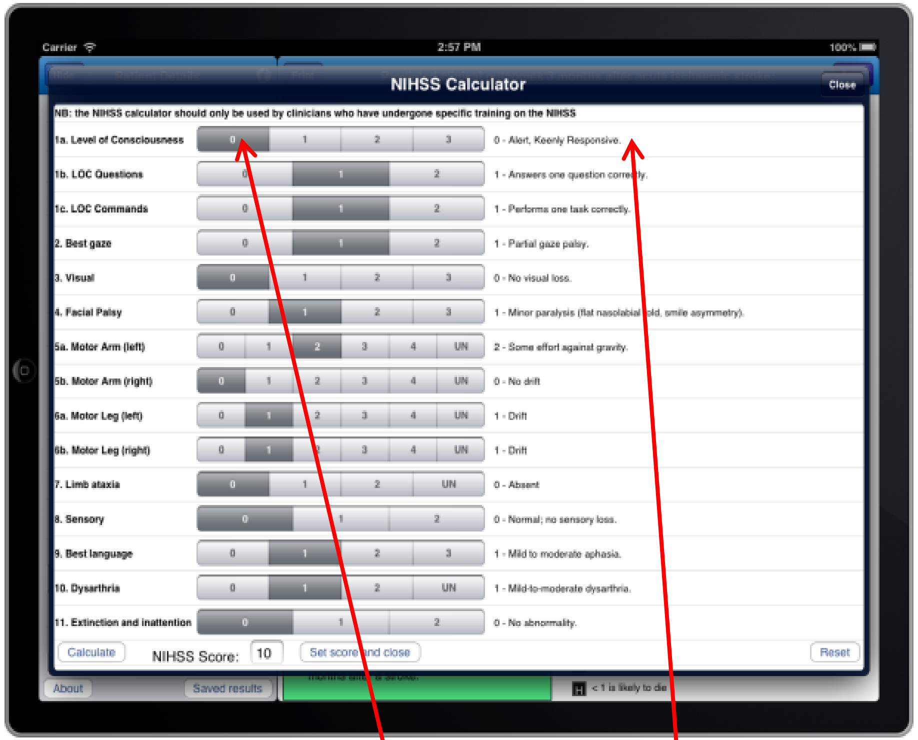

An electronic version of the NIHSS (used by trained qualified stroke physicians to assess the severity and extent of impairments due to stroke) is embedded in COMPASS.

By selecting the **numerical value** for the corresponding category in the NIHSS a **textual description** for the value selected is displayed to the right of the screen

Timeline Graph

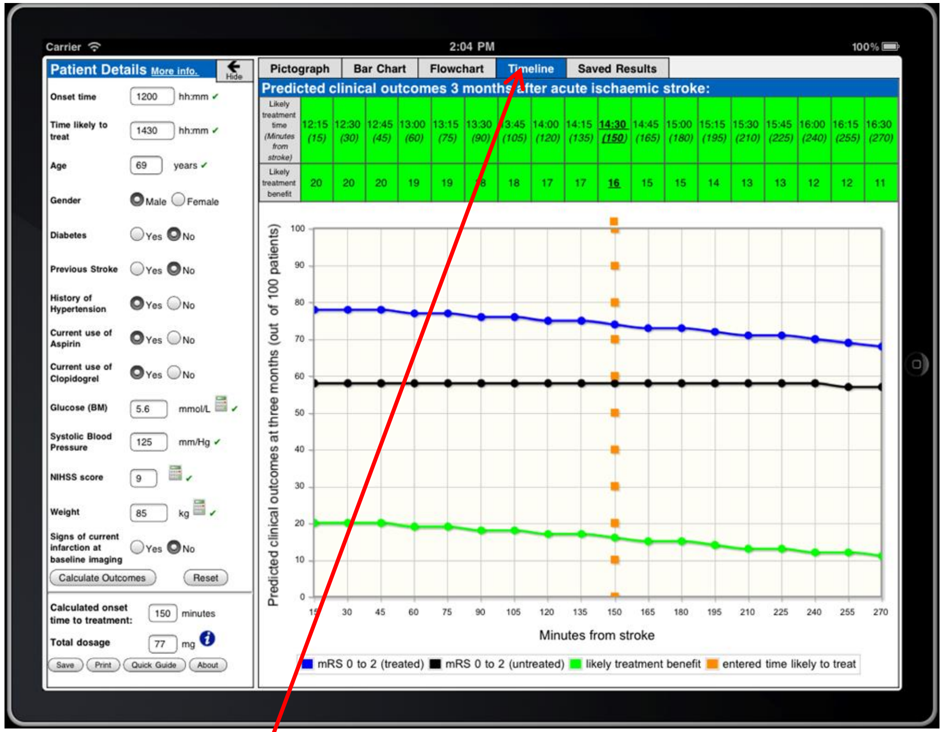

After patient details have been populated and the treating clinician has selected ‘calculate outcomes, by selecting the **timeline tab** the following information is displayed:

- Numerical values (out of 100 patients) for predicted net benefit (mRS 0 to 2) from thrombolytic treatment as a function of increasing stroke onset time to treatment in 15 minutes intervals
- The same information is shown in the line graph (green line)

Save functionality

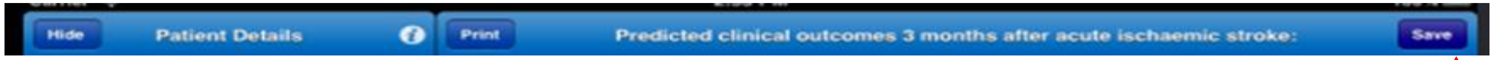

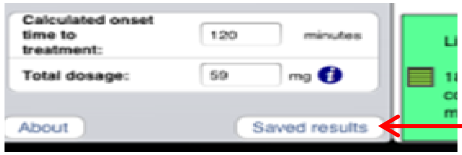

Tap here to open and view saved patient details and related predicted clinical outcomes.

Tap here to save entered patient details and predicted clinical outcomes

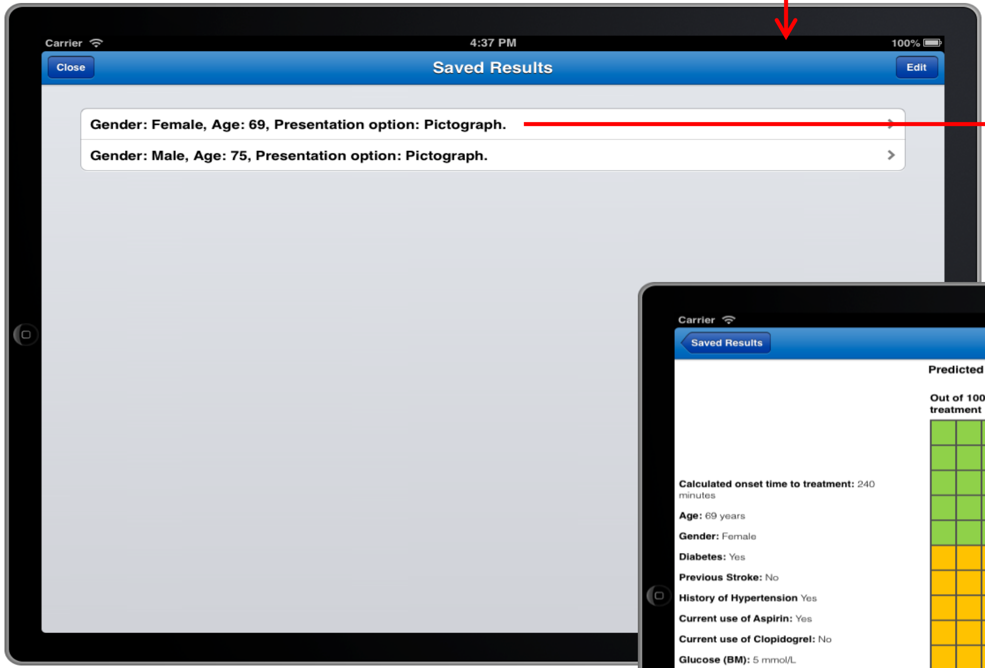

Select the desired save result to view – see below

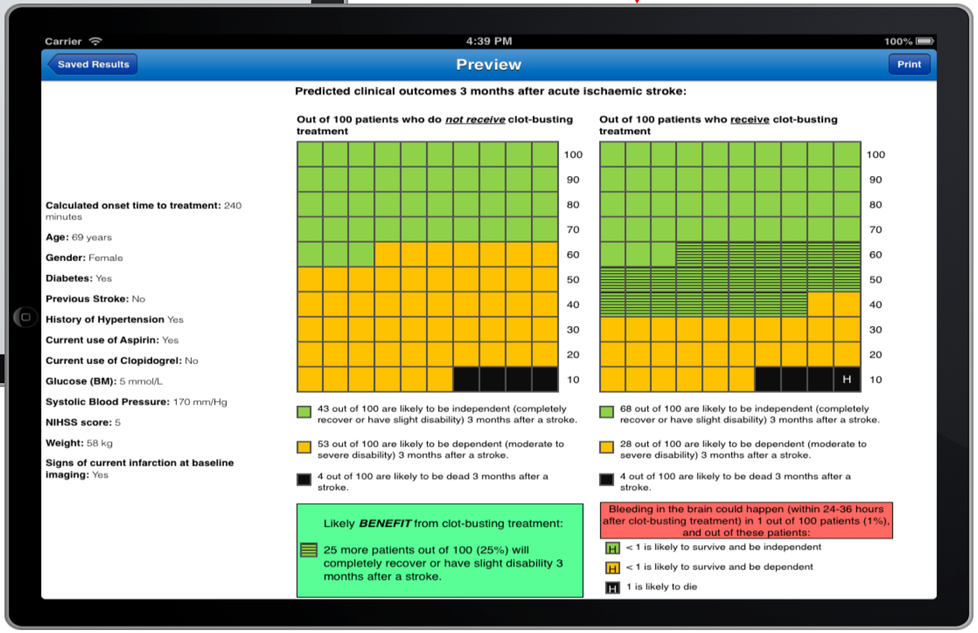

Print functionality

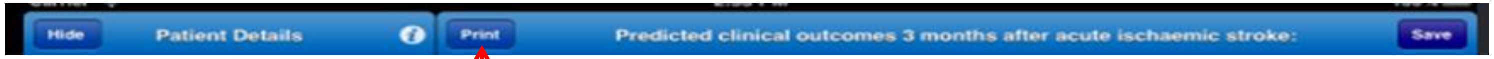

Tap print for a hard copy of predicted clinical outcomes (entered or saved) in either monochrome (black and white or colour). NB: wireless printer recommended

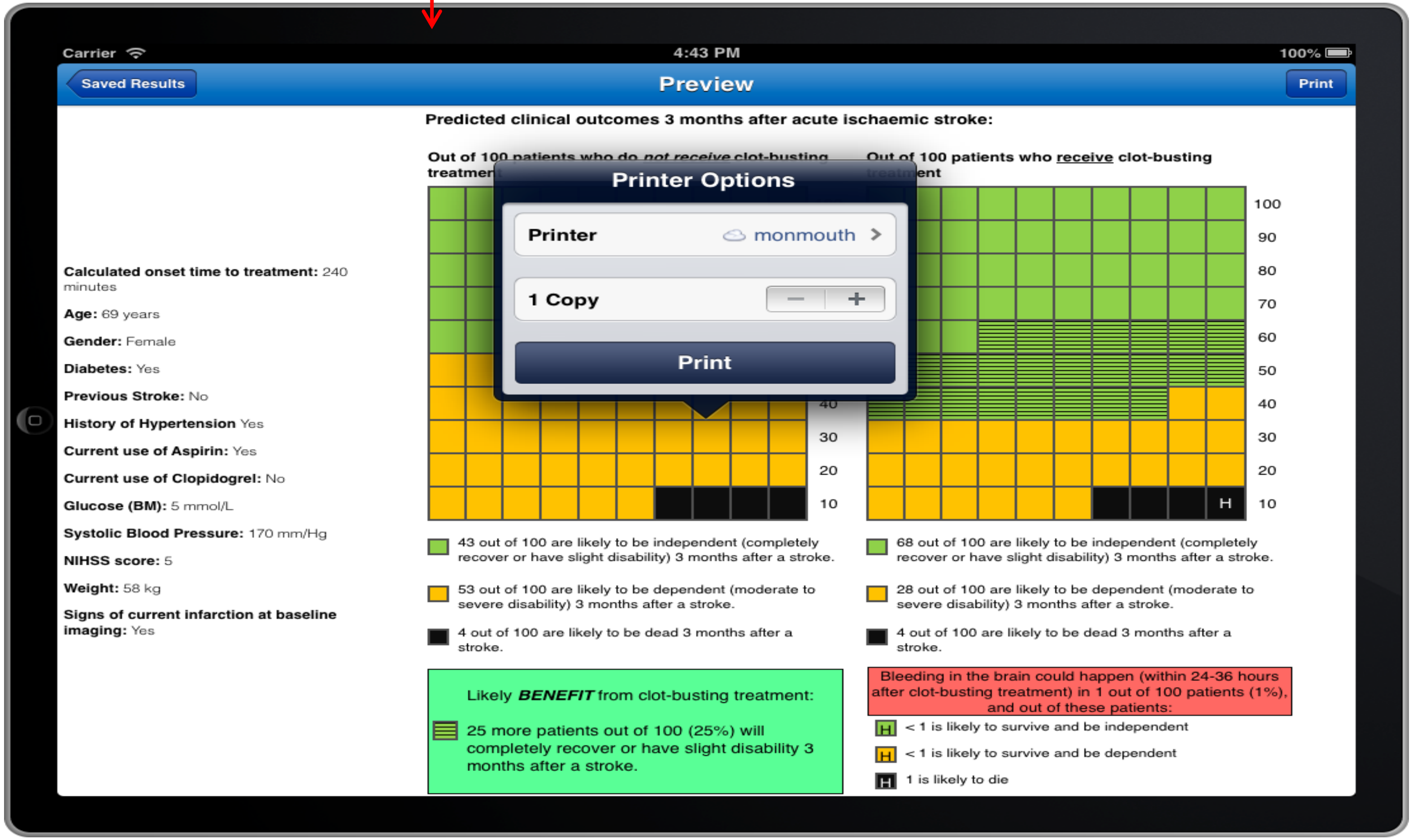

NB: the save and print functionality can be used to facilitate case review within clinical meetings
